# Case Report: Medullary carcinoma of the pancreas with *MLH1* promoter hypermethylation, induced deficient mismatch repair, successfully treated with an immune checkpoint inhibitor

**DOI:** 10.3389/fonc.2025.1551038

**Published:** 2025-03-27

**Authors:** Koushiro Ohtsubo, Shigeki Sato, Hiroyuki Sakaguchi, Hiroshi Kotani, Akihiro Nishiyama, Kaname Yamashita, Seiji Yano, Fumihito Toshima, Dai Inoue, Toshifumi Gabata, Hiroko Ikeda, Atsushi Watanabe, Kenji Notohara, Takao Fujisawa, Yoshiaki Nakamura, Takayuki Yoshino, Kunio Miyake, Kazuhiro Miwa, Shinji Takeuchi

**Affiliations:** ^1^ Department of Medical Oncology, Kanazawa University Hospital, Kanazawa, Japan; ^2^ Department of Respiratory Medicine, Kanazawa University Hospital, Kanazawa, Japan; ^3^ Department of Radiology, Kanazawa University Hospital, Kanazawa, Japan; ^4^ Division of Human Pathology, Kanazawa University Hospital, Kanazawa, Japan; ^5^ Division of Clinical Genetics, Kanazawa University Hospital, Kanazawa, Japan; ^6^ Department of Anatomic Pathology, Kurashiki Central Hospital, Kurashiki, Japan; ^7^ Department of Head and Neck Medical Oncology, National Cancer Center Hospital East, Kashiwa, Japan; ^8^ International Research Promotion Office, National Cancer Center Hospital East, Kashiwa, Japan; ^9^ Translational Research Support Office, National Cancer Center Hospital East, Kashiwa, Japan; ^10^ Department of Gastrointestinal Oncology, National Cancer Center Hospital East, Kashiwa, Japan; ^11^ Department of Epidemiology and Environmental Medicine, Interdisciplinary Graduate School of Medicine and Engineering, University of Yamanashi, Chuo, Japan; ^12^ Internal Medicine, Komatsu Municipal Hospital, Komatsu, Japan

**Keywords:** medullary carcinoma of the pancreas, deficient mismatch repair, microsatellite instability, *MLH1* hypermethylation, immune checkpoint inhibitor, pembrolizumab

## Abstract

We report the case of a 75-year-old woman with a pancreatic body mass. Pathological findings from endoscopic ultrasonography-guided fine-needle aspiration revealed medullary carcinoma of the pancreas (MCP). Deficient mismatch repair (dMMR) and high microsatellite instability (MSI-H) were identified through immunohistochemistry and next generation sequencing, respectively. While immunohistochemistry suggested MLH1 abnormality, no *MLH1* mutation was; hypermethylation of the *MLH1* promoter was later confirmed via bisulfite sequencing. The patient initially received nab-paclitaxel plus gemcitabine, achieving tumor shrinkage. Upon tumor regrowth, she was treated with the anti-programmed cell death-1 immune checkpoint inhibitor (ICI) pembrolizumab, which resulted in significant tumor reduction. This is the first case report of MCP with dMMR/MSI-H due to *MLH1* promoter hypermethylation, effectively treated with an ICI.

## Introduction

Pancreatic cancer (PC) is a highly fatal disease with a 5-year survival rate of approximately 10% and is becoming an increasingly common cause of cancer-related mortality (1, 2). Approximately 95% of PCs are exocrine cell tumors, with ductal adenocarcinomas being the most prevalent subtype (3).

Medullary carcinoma of the pancreas (MCP) is a rare subtype of pancreatic ductal adenocarcinoma recognized in the current World Health Organization classification (4). Pathologically, MCP is characterized as a poorly differentiated carcinoma with limited gland formation, sheets and nests with pushing borders, syncytial growth patterns, and often abundant tumor-infiltrating lymphocytes (4). Although MCP has frequently been associated with deficient mismatch repair (dMMR) and high microsatellite instability (MSI-H) (5–12), most cases are linked to Lynch syndrome, with *MLH1* promoter hypermethylation being reported only anechdotally (9). Furthermore, the effectiveness of immune checkpoint inhibitors (ICI) in MCP remains unclear.

We report a rare case of MCP with dMMR/MSI-H due to *MLH1* promoter hypermethylation, successfully treated with immunotherapy.

## Case report

A 75-year-old woman presented to a local hospital complaining of lumbago. Abdominal computed tomography (CT) revealed a large pancreatic body mass, and she was referred to our hospital for further evaluation and treatment.

**Figure 1:**
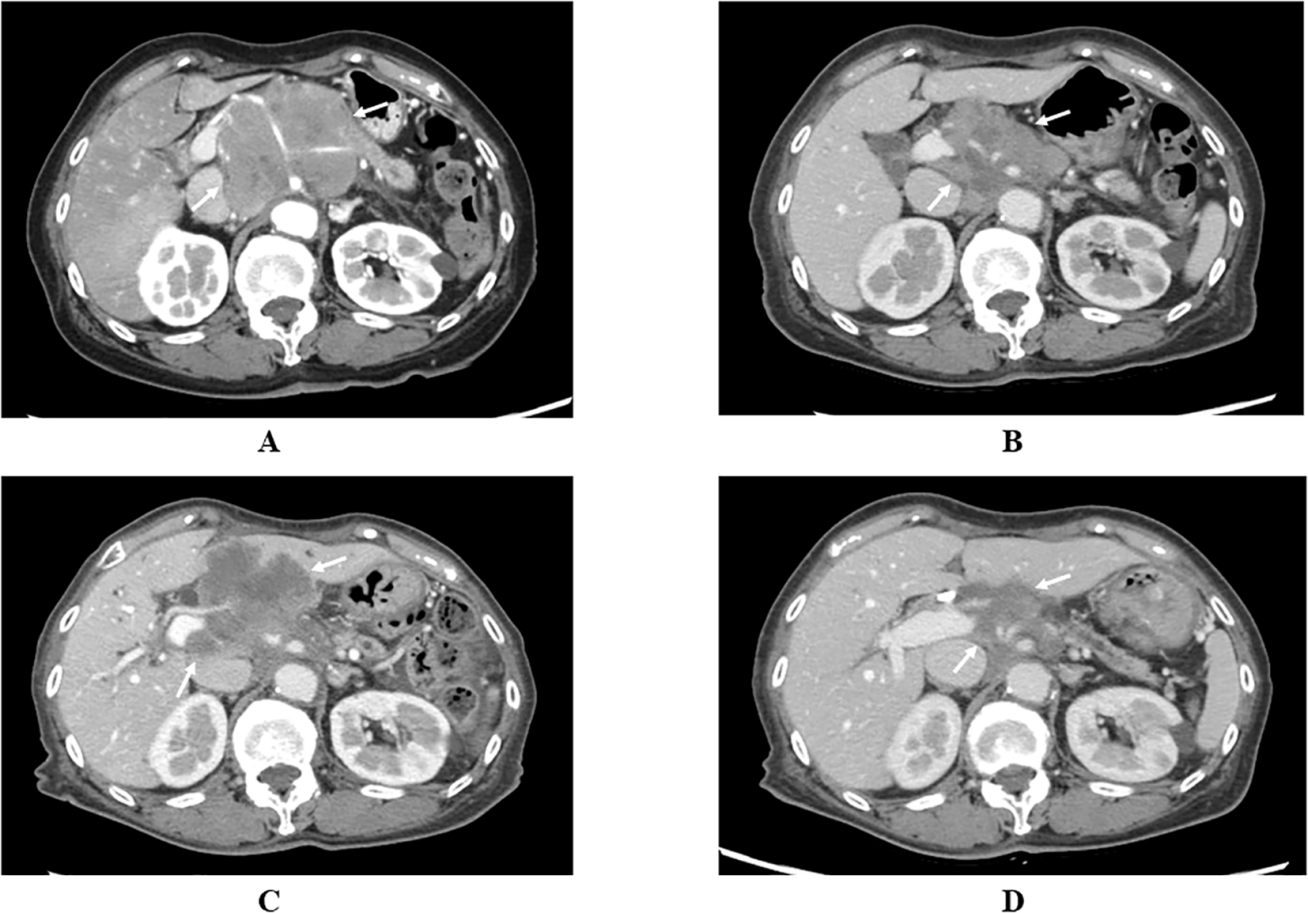
**(A)** Contrast-enhanced abdominal computed tomography showing a 77-mm hypovascular mass with celiac artery invasion in the pancreatic body (white arrows). **(B)** Significant reduction in pancreatic tumor size, indicating partial response, after two months of treatment with nab-paclitaxel plus gemcitabine (white arrows). **(C)** Enlargement of the pancreatic tumor with obstructive jaundice after six months of treatment (white arrows). **(D)** Marked reduction in pancreatic tumor size, nearly achieving partial response, four months after initiating pembrolizumab treatment (white arrows).

Her medical history included ischemic heart disease and uterine myoma. Her family history was significant for gastric cancer in an elder brother and breast cancer in two elder sisters. She neither smoked, nor drank alcohol. Pancreatic enzyme levels were elevated, including lipase (54 IU/L; reference range: 11–53 IU/L), elastase I (329 ng/dL; reference range: <300 ng/dL), and trypsin (716 ng/mL; reference range: 110-550 ng/mL), while amylase remained normal. Among tumor markers, carcinoembryonic antigen (CEA), carbohydrate antigen 19-9 (CA 19-9), and Duke pancreatic monoclonal antigen type 2 (DUPAN-2) were within normal ranges, but cytokeratin-19 fragment (CYFRA 21-1) was elevated to 4.1 ng/mL (reference range: <3.5 ng/mL). Contrast-enhanced abdominal CT revealed a 77-mm hypovascular mass in the pancreatic body with celiac artery invasion (**Figure 1A**), but no definitive distant metastases were identified. The pancreatic tumor was classified as T4N0M0, stage III according to the Union for International Cancer Control (UICC) 8^th^ edition.

**Figure 2:**
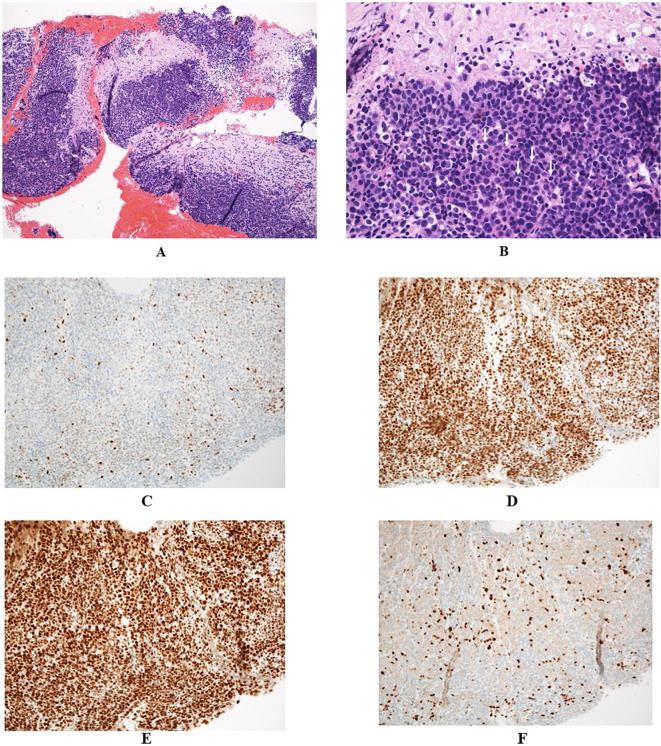
**(A, B)** Pathological findings revealed poorly differentiated carcinoma with syncytial growth, tumor-infiltrating lymphocytes (examples marked with white arrows), formation of sheets and nests, and pushing borders, suggesting medullary carcinoma (**A**:×10, **B**:×40). **(C–F)** Immunohistochemical findings demonstrated deficient mismatch repair proteins: MLH1 **(C)** and PMS2 **(F)**, and proficient MSH2 **(D)** and MSH6 **(E)**, suggesting an *MLH1* gene abnormality (**C–F**:×20).

Endoscopic ultrasonography (EUS) demonstrated a low-echoic, lobulated mass, and EUS-guided fine-needle aspiration (EUS-FNA) was performed using a 19-gauge needle. Pathological examination identified medullary carcinoma, characterized by solid sheets of carcinoma cells with minimal stroma (**Figure 2A**) and abundant tumor-infiltrating lymphocytes (**Figure 2B**). Staining for mismatch repair (MMR) proteins revealed loss of MLH1 and PMS2 expression, with retained MSH2 and MSH6, indicating deficient mismatch repair (dMMR) due to MLH1 dysfunction (**Figures 2C–F**). Although solid medullary neoplasms of the pancreas were raised as differential diagnosis, neuroendocrine neoplasms were excluded based on the negative immunohistochemical staining for synaptophysin, chromogranin A, and insulinoma-associated protein 1 (INSM1). Additionally, Bcl-10 immunostaining for acinar cell carcinoma and Epstein-Barr virus (EBV)-encoded RNA (EBER) *in situ* hybridization for EBV-related poorly differentiated carcinoma were also negative, further supporting the diagnosis of pancreatic medullary carcinoma.

**Figure 3:**
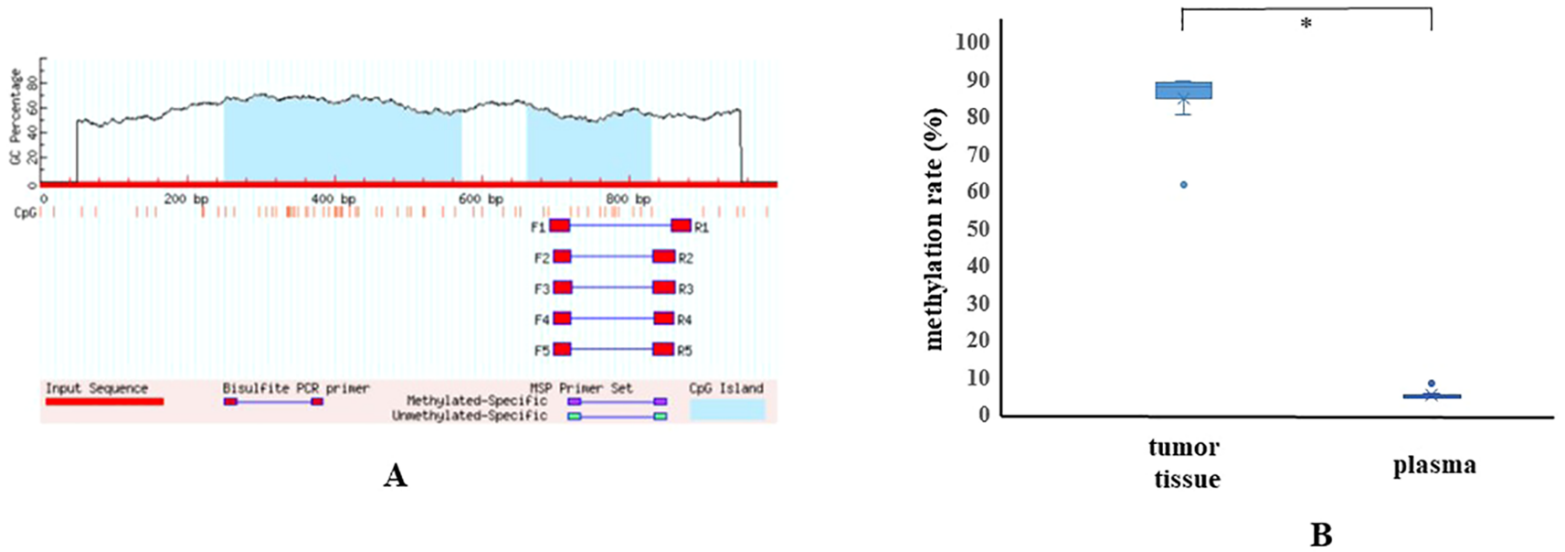
**(A)** CpG sites in the *MLH1* gene are shown. Methylation analysis of *MLH1* was performed at 11 CpG sites using 5 primers by bisulfite sequencing. **(B)** The methylation rate of the *MLH1* promoter region in pancreatic tumor tissue (61.4–89.5%, average: 84.5%) was significantly higher than that in plasma (4.2–10.4%, average: 5.4%) (p=0.0001; Mann-Whitney U test). The methylation rate in normal pancreatic tissue could not be analyzed due to insufficient material.

Genetic analysis was performed using both pancreatic tumor tissue and blood samples under MONSTAR-SCREEN-2, a component of the Cancer Genome Screening Project for Individualized Medicine in Japan (SCRUM-Japan) (13). Tumor tissue next-generation sequencing (NGS) was performed using MI Profile^®^ (Caris Life Sciences, Phoenix, AZ), while plasma and buffy coat NGS was performed using Caris Assure^®^ (Caris Life Sciences, Phoenix, AZ). No abnormalities were detected in the four major driver genes of pancreatic cancer (*KRAS, TP53, CDKN2A, SMAD4).* However, MSI-H and high tumor mutational burden (TMB-H) were identified in both tumor tissue (31 Mut/Mb) and plasma (19 Mut/Mb) (**Tables 1A, B**). Germline analysis detected no pathogenic variants in the MMR genes. The absence of somatic or germline mutations in the MMR genes suggested an epigenetic alteration of *MLH1*. Bisulfite sequencing confirmed hypermethylation of the *MLH1* promoter in tumor tissue (**Figure 3**). Additionaly, an *E-cadherin (CDH1)* mutation with high variant allele frequency was identified in both pancreatic tumor tissue and plasma (**Tables 1A, B**).

**Table 1:** Genes tested with pathogenic or likely pathogenic alterations.

A. Pancreatic tumor tissue				
Genes	Exon	DNA alterations	Protein alterations	Variant frequency (%)
AXIN1	10	c.2385dupC	Y796fs	41
	6	c.1523delG	G508fs	14
	4	c.1034delC	P345fs	16
CDH1	5	c.569A>G	Y190C	86
CREBBP	16	c.3250delA	I1084fs	13
ERBB2 (Her2/Neu)	19	c.2264T>C	L755S	45
ERBB3	9	c.1064C>T	T355I	41
KMT2D	31	c.6783 _6798 delins8	V2263fs	39
MGA	3	c.1879C>T	R627*	44
MSH3	7	c.1148delA	K383fs	56
NF1	18	c.2033dupC	I679fs	35
PPP2R1A	10	c.1252C>T	R418W	36
SDHA	15	c.1974delG	P659fs	10
STAT3	21	c.1981G>T	D661Y	40
MSI-high				
TMB-high (31 mut/Mb)				

B. Plasma				
Genes	Protein alterations	Variant frequency (%)
ARID1A	S536fs	16
AXIN1	E640fs	3.5
	G265fs	2.2
	G508fs	9
CASP8	P411L	4.3
CDH1	Y190C	32
ERBB2 (HER2/NEU)	L755S	15
ERBB3	T355I	14
KMT2D	A2268fs	9
MAP2K1 (MEK1)	F53L	1.2
MGA	R627*	11
MSH3	K383fs	32
NF1	I679fs	13
PPP2R1A	R418W	16
STAT3	D661Y	11
MSI-high				
Blood TMB 19 mut/Mb				

**Figure 4:**
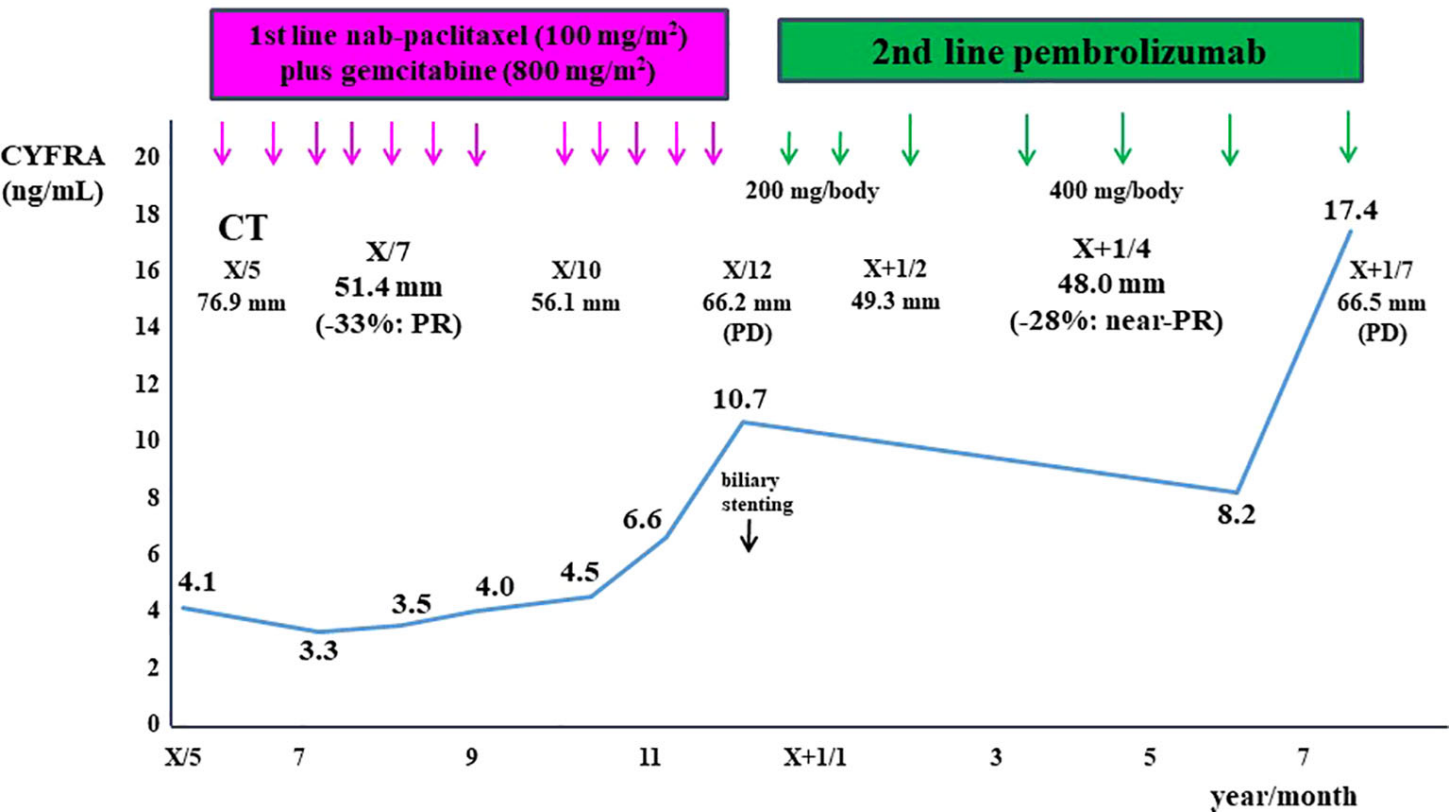
Clinical course of this patient is shown. The patient was initially treated with nab-paclitaxel plus gemcitabine, resulting in a partial response after two months. However, tumor regrowth occurred six months after treatment initiation. The patient was subsequently treated with pembrolizumab, achieving near- partial response after four months. However, tumor regrowth occurred seven months after the initiation of pembrolizumab. The cytokeratin-19 fragment (CYFRA 21-1) levels closely correlated with tumor size.

The patient was initially treated with nab-paclitaxel plus gemcitabine, resulting in dramatic tumor shrinkage and a partial response (PR) after two months (**Figure 1B**). However, six months after initiating treatment, tumor regrowth occurred, accompanied by obstructive jaundice (**Figure 1C**). Endoscopic placement of a biliary plastic stent was performed, followed by treatment with the anti-programmed cell death-1 (PD-1) ICI pembrolizumab. The tumor significantly reduced in size again, achieving near-PR after four months (**Figure 1D**). Although tumor shrinkage was maintained for two months, tumor regrowth occurred seven months after the initiation of pembrolizumab, leading to treatment discontinuation (**Figure 4**).

## Discussion

MCP is a rare histological subtype of PC (4, 14), first described by Goggins et al. in 1998 (5). Pathologically, MCP is characterized by poorly differentiated carcinoma with pushing borders, limited gland formation, and syncytial growth of tumor cells, often accompanied by abundant tumor-infiltrating lymphocytes (4). MCP is associated with a relatively favorable prognosis (6, 7, 15, 16). While MCP has been reported in EBV-associated carcinomas with lymphoepithelioma-like features (6), EBV involvement was excluded in this case due to a negative EBER *in situ* hybridization.

Carcinomas with dMMR/MSI-H are frequently observed in endometrial, gastric, small bowel, and colorectal cancers (CRCs) (17, 18). In CRCs, Alexander et al. identified medullary carcinoma, intraepithelial lymphocytosis, and poor differentiation as key features distinguishing MSI-H from microsatellite-stable (MSS) cancers with high specificity (19). Although the overall frequency of dMMR/MSI-H in PCs is extremely low (around 1–2%) (10, 17, 18, 20), it is relatively high in MCPs (5, 6, 10, 12). A systematic review of 34 studies involving 8,323 patients by Luchini et al. (10) reported that dMMR/MSI-H PCs are rare but are strongly associated with medullary or mucinous/colloid histology and a *KRAS/TP53* wild-type molecular background.

Lynch syndrome, also known as hereditary nonpolyposis colorectal cancer syndrome, is an autosomal dominant disorder caused by germline mutations in any of the four genes involved in the MMR process: *MLH1, MSH2, MSH6*, and *PMS2* (21). The most common malignancies in Lynch syndrome patients are CRCs and endometrial cancers, with penetrance rates of 50–80% and 25–60%, respectively (22). PC associated with Lynch syndrome was firstly reported in 1985 (23). Lynch syndrome is associated with an approx. 8.6-fold increased risk of developing PC (24) and has been identified in patients with MCP (6, 8). Given that MCP generally exhibits the histological features of a poorly differentiated carcinoma, we propose that poorly differentiated carcinomas reported in Lynch syndrome patients (8, 10) are highly likely to represent MCP (25, 26).

In this case, hypermethylation of *MLH1* is estimated to be main cause of tumorigenesis, as bisulfite sequencing validated *MLH1* hypermethylation while no mutations in MMR genes were detected. Hypermethylation serves as an alternative mechanism for the genetic silencing of *MLH1*. In CRC, it has been documented that dMMR/MSI-H is induced by hypermethylation of the *MLH1* promoter region in over 75% of dMMR carcinomas, while germline mutations associated with Lynch syndrome account for less than 25% (27). Although the reported prevalence of *MLH1* hypermethylation ranges from 0% to 54% (28–30), its precise role in *MLH1* gene silencing remains unclear. Only one case of MCP harboring *MLH1* promoter hypermethylation has been reported, identified using methylation-specific PCR (9).

In most PC patients, dMMR/MSI-H is routinely investigated using PCR and/or MMR immunohistochemistry rather than NGS (10). However, germline and methylation analyses have been scarcely performed. Consequently, it remains unclear whether genetic (somatic or germline mutations) or epigenetic mechanisms predominate in the development of MCP.

The typical genetic pattern of MCP is believed to involve dMMR/MSI-H and wild-type *KRAS* (5, 6). However, dMMR/MSI-H has been detected in only 22% (4/18) of MCP cases, with the majority being MSS (5, 6, 31). Mutations in the proofreading domain of *polymerase epsilon (POLE)* result in DNA repair deficiency characterized by MSS and an ultramutated phenotype (32). A case of MCP with a somatic *POLE* mutation and MSS has been reported, where the *POLE* mutation and resulting hypermutation were proposed as an altenative genetic backgound for MCP distinct from dMMR/MSI-H (33).

In a recent report, none of the six patients with MSI-H PC who received chemotherapy achieved a clinical response, including four patients treated with nab-paclitaxel plus gemcitabine (20). However, the patient was initially treated with nab-paclitaxel plus gemcitabine, resulting in a PR. Conversely, although the objective response rate of the anti-PD-1 ICI, pembrolizumab, in patients with previously treated advanced noncolorectal dMMR/MSI-H cancers has been reported as 34.3% (80 of 233 patients), the response rate in PC was only 18.2% (4 of 22 patients) in the Phase II KEYNOTE-158 study (34). Additionally, objective responses were observed in 29% (30 of 102 patients) of the tissue TMB-H group treated with pembrolizumab (35). In the present case, both dMMR/MSI-H and TMB-H (31 Mut/Mb) were identified in pancreatic tumor tissue, and the tumor size was further reduced following secondary treatment with pembrolizumab, resulting in nearly a PR. To the best of our knowledge, this is the first case report demonstrating the efficacy of immunotherapy in a patient with MCP. However, tumor regrowth occurred seven months after the initiation of pembrolizumab. Acquired resistance to anti-PD-1 ICI in patients with melanoma has been reported to be associated with defects in the pathways involving interferon-receptor–associated *Janus kinase 1* (*JAK1*) or *Janus kinase 2* (*JAK2*), as well as in the antigen-presenting protein *beta-2-microglobulin* (*B2M*) (36). Further investigation is necessary to clarify acquired resistance to anti-PD-1 ICI in patients with MCP.

In the present case, a *CDH1* mutation with a high variant allele frequency was detected, and a germline pathogenic variant was suspected based on NGS analysis. Additionally, the presence of gastric cancer in the patient’s elder brother and breast cancers in two elder sisters aligns with the phenotype associated with *CDH1* mutation. It has been reported that *CDH1* germline mutations are linked to hereditary diffuse gastric cancer and lobular breast cancer (37–39). A recent report also described a relationship between *CDH1* germline mutations and colorectal signet-ring cell cancer; however, the development of pancreatic cancer has been rarely documented (38). Furthermore, since no germline variants were detected in buffy coat analysis, we determined that the *CDH1* mutation was somatic. Therefore, in the current case, the *CDH1* mutation is unlikely to be significantly associated with the development of MCP.

In conclusion, we present a rare case of MCP with dMMR/MSI-H attributed to *MLH1* promoter hypermethylation, successfully treated with immunotherapy. This case highlights the importance of testing for dMMR/MSI-H in MCP cases to assess their potential responsiveness to immunotherapy. Further research is needed to elucidate the pathogesesis of MCP.

## Data Availability

The original contributions presented in the study are included in the article/supplementary material. Further inquiries can be directed to the corresponding author.
